# Soft tissue cell adhesion to titanium abutments after different cleaning procedures: Preliminary results of a randomized clinical trial

**DOI:** 10.4317/medoral.19329

**Published:** 2013-10-13

**Authors:** Luigi Canullo, David Peñarrocha-Oltra, Silvia Marchionni, Leticia Bagán, Maria A. Peñarrocha-Diago, Costanza Micarelli

**Affiliations:** 1 Private practice in Rome, Italy; 2 Oral Surgery Unit, Stomatology Department, Faculty of Medicine and Dentistry, University of Valencia, Spain; 3 Department of Oral Sciences, Laboratory of Microscopy, Alma Mater Studiorum, University of Bologna, Italy; 4 Collaborator of Oral Medicine, Stomatology Department, Faculty of Medicine and Dentistry, University of Valencia, Spain; 5 Full Professor of Oral Surgery, Stomatology Department, Faculty of Medicine and Dentistry, University of Valencia, Spain; 6 Private practice in Rome, Italy

## Abstract

Objectives: A randomized controlled trial was performed to assess soft tissue cell adhesion to implant titanium abutments subjected to different cleaning procedures and test if plasma cleaning can enhance cell adhesion at an early healing time. Study Design: Eighteen patients with osseointegrated and submerged implants were included. Before re-opening, 18 abutments were divided in 3 groups corresponding to different clinical conditions with different cleaning processes: no treatment (G1), laboratory customization and cleaning by steam (G2), cleaning by plasma of Argon (G3). Abutments were removed after 1 week and scanning electron microscopy was used to analyze cell adhesion to the abutment surface quantitatively (percentage of area occupied by cells) and qualitatively (aspect of adhered cells and presence of contaminants). Results: Mean percentages of area occupied by cells were 17.6 ± 22.7%, 16.5 ± 12.9% and 46.3 ± 27.9% for G1, G2 and G3 respectively. Differences were statistically significant between G1 and G3 (p=0.030), close to significance between G2 and G3 (p=0.056), and non-significant between G1 and G2 (p=0.530). The proportion of samples presenting adhered cells was homogeneous among the 3 groups (p-valor = 1.000). In all cases cells presented a flattened aspect; in 2 cases cells were less efficiently adhered and in 1 case cells presented filipodia. Three cases showed contamination with cocobacteria. Conclusions: Within the limits of the present study, plasma of Argon may enhance cell adhesion to titanium abutments, even at the early stage of soft tissue healing. Further studies with greater samples are necessary to confirm these findings.

** Key words:**Connective tissue, dental abutments, randomized controlled trial, clinical research, glow discharged abutment, plasma cleaning.

## Introduction

Dental implants pierce the oral mucosa and establish a transmucosal connection between the external environment and the inner parts of the body. The early formation of a long-standing biological barrier capable of preventing bacterial penetration through this transmucosal piercing is most important for long-term implant success. This soft tissue barrier faces the titanium abutment surface, and it can be divided into 2 zones; one marginal zone that harbors a junctional epithelium and one more apical zone comprised of a fiber rich connective tissue (1,2). The quality of this mucosal attachment is influenced by the properties of the implant components that are placed in contact with the soft tissues (3).

After technical procedures, presence of contaminants (mostly Titanium wear micro-particles, Carbon and Aluminum traces due to lubricant used during customization) on the abutment surface can be found, even after the usual cleaning steps (steaming) (4). Such debris, present at the titanium/connective-bone tissues interface, minimize the soft tissue adhesion and could deleteriously influence the inflammatory response of the peri-implant tissues (5).

Although specific protocols have been proposed, it is proven to be difficult to effectively clean contaminated titanium surfaces (6,7). Vezeau et al. (8) performed an in vitro study to investigate various cleaning and sterilization regimens for the removal of biological debris to support reattachment of subgingival connective tissue. Titanium discs were sterilized by ultraviolet light or steam autoclaving both with and without previous treatment with plasma of Argon. Cell attachment was significantly reduced by autoclaving, while sterilization with ultraviolet light resulted in relatively high levels of cell attachment. Plasma cleaning, applied before ultraviolet light treatment, enhanced surface energetics but did not affect cell attachment and spreading. In more recent “in vitro” studies, plasma of Argon cleaning treatment was demonstrated to have a double effect on titanium abutments: removal of pollutions following customization and increase of cell adhesion (9,10).

The aim of this “in vivo” randomized controlled trial was to assess soft tissue cell adhesion to titanium abutments subjected to different cleaning procedures - no treatment (as they come from the industry), cleaning by steam after laboratory customization and cleaning by plasma of Argon – at an early healing time. The null hypothesis of the study was that the cleaning procedure applied to implant abutments has no effect on soft tissue cell adhesion at an early healing time.

The article was written following the CONSORT statement for improving the quality of RCTs (11).

## Material and Methods

Study design and patient selection

A preliminary prospective, match-paired, triple-blinded randomized controlled clinical trial was performed following the principles outlined in the Declaration of Helsinki. All procedures were approved by the local Ethical Committee of the University of Valencia, and patients were required to sign a consent form. Patients were recruited at the Oral Surgery Unit of the University of Valencia (Spain) during February and March 2013.

Eighteen patients in general good health scheduled for implant-supported restorations were required for this pilot study. Sites with acute infection or requiring regenerative procedures, patients < 18 years of age, with smoking habit (>10 cig/day), with Full Mouth Plaque Score and Full Mouth Bleeding Score > 25 %, pregnant and lactating or with history of bisphosphonates were excluded. Each patient presented one submerged and osseointegrated Global implant (Sweden & Martina, Padua, Italy) .

Eighteen screw-retained healing abutments, especially designed for the study, were divided in 3 groups and submitted to different cleaning processes: no treatment (as they come from the industry, G1), laboratory customization and cleaning by steam (G2), cleaning by plasma of Argon (G3).

Cleaning processes.

G2 abutments underwent cleaning by steam, performed for 5 sec at 4atm 4 MPa (VAP 1, Zhermark, Cologne, Germany).

G3 abutments underwent argon plasma treatment in a plasma reactor (Diener Electronic GmbH, Jettingen, Germany). The treatment conditions were 75 W of power and -10MPa of pressure for 12 minutes.

Cleaning processes were performed immediately before second surgery.

All abutments were conveyed to the surgeon in a sterile envelope.

Randomization

Immediately before reopening patients included in the study were randomly assigned to one of the three treatments.

Random assignment of the implant sites to the groups within each patient was performed according to predefined randomization tables. A balanced random permuted block approach was used to prepare the randomization tables to prevent an unequal balance between the three groups. A statistician generated the allocation sequence and assigned participants to their groups. Assignment was performed using sealed envelopes. Participants were informed about the different treatments, but blinded to the assignment.

Sample obtainment, processing and examination

Second surgeries were performed by a blinded operator 10 weeks after implant insertion. After local anesthesia, minimally invasive flaps were performed and abutments were screwed at 32N/cm. One week after the second surgeries, abutments were disconnected and fixed in 4% glutaraldehyde in 0.2M sodium-cacodilate buffer. The specimens were then washed in 0.1M sodium cacodilate buffer, dehydrated in graded alcohol, air dried and gold/palladium-coated (Quorum Emitech Sc7620).

**Figure 1:**
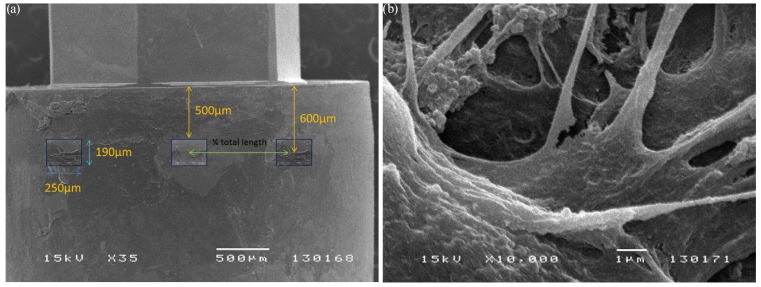
Examination of the abutment surface using SEM. (a) Standardization method used to determine the 250 x 190 µm observation fields. (b) Image with x10000 magnification showing adhered fibroblasts with flattened aspect in the G3 sample.

The specimens were then examined using a scanning electron microscope (SEM) (JSM-5200; JEOL, Tokyo, Japan) and images captured with a software package (Semafore, JEOL, Sweden). Images of each specimen were obtained according to a standardized study design: each observation point was predetermined following a grid and rules formerly decided by the researcher, eliminating the bias caused by the investigator behavior. The methods allowed repeated observations with 100% repeatability in finding the same observation fields when a 250 x 190 µm frame was adopted. For each specimen, 3 equidistant images with a frame of 250 x 190 µm and a magnification of 500x were captured at 500 µm away from the implant/abutment junction (Fig. 1). Additionally, images with higher magnification were captured in the most interesting sites (Fig. 1).

Assessed outcomes

A blinded histologist performed a quantitative and a qualitative evaluation using the obtained images. The quantitative study of scanning electron micrographs was done semi-automatically using three B/W images per case at the same magnification. The macro was implemented with ImageJ Program (v1.46 rsbweb.nih.gov/ij). The primary variable assessed was the percentage of the total area occupied by cells. Presence or absence of cells (secondary variable) was assessed semi-quantitatively as a dichotomous variable. Moreover, the histologist evaluated the aspect of the adhered cells and the presence of contaminants.

Statistical analysis

A descriptive analysis was performed separately for the 3 groups including mean, standard deviation, median, maximum and minimum for the percentage of area occupied by cells. A comparative analysis was performed using an “intention-to-treat” approach and applying non-parametric tests due to the reduced sample size. Kruskal-Wallis test was used to assess differences in the percentage of area occupied by cells between the 3 treatment groups. Mann-Whitney test was then used to assess between which pairs of groups differences were statistically significant. A power analysis was performed for a Mann-Whitney test, using an alpha value set at 0.05 and considering a size effect of 0.8, to detect significant differences between group pairs. Fisher’s exact test was used to compare the proportions of presence/absence of cells taking 2 groups at a time. Statistical analysis was performed with SPSS 20.0 software (SPSS Inc., Chicago, IL) using an alpha value set at 0.05. A biostatistician with expertise in dentistry analyzed the data without knowing the group assignment.

## Results

**Figure 2:**
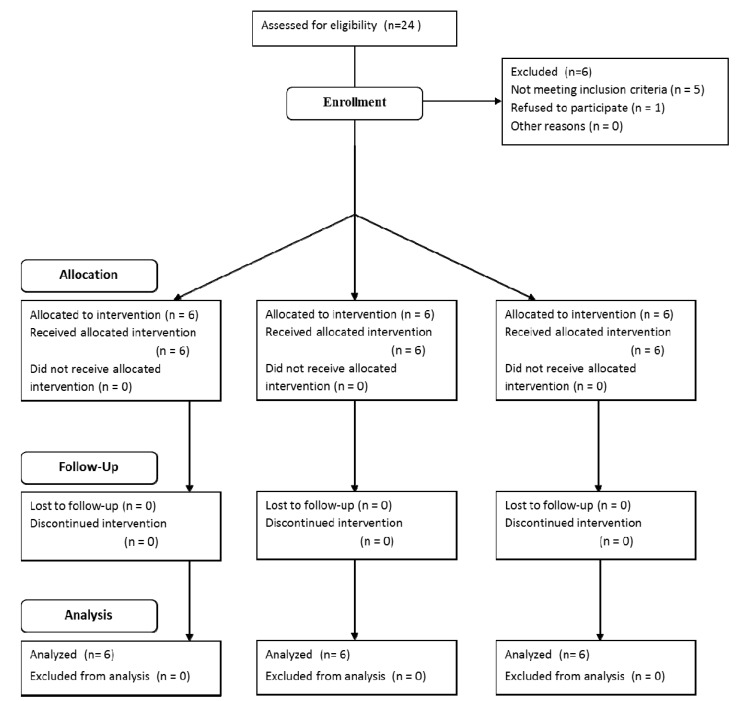
Flow chart of participants’ enrollment in the study.

Twenty-four consecutive patients were initially considered to be included in the study. Five patients were excluded for not fulfilling the required criteria (4 smoked > 10 cigarettes/day and 1 had been treated with bisphosphonates) and 1 patient refused to participate. The final sample consisted of 18 patients (7 men and 11 women) between 36 and 68 years of age (mean age 51.5 years). Six patients were allocated to each group. All patients fulfilled the 1 week follow-up and were analyzed (Fig. 2). Surgeries and post-operative healing periods were without any complication or side effect for all patients.

**Figure 3:**
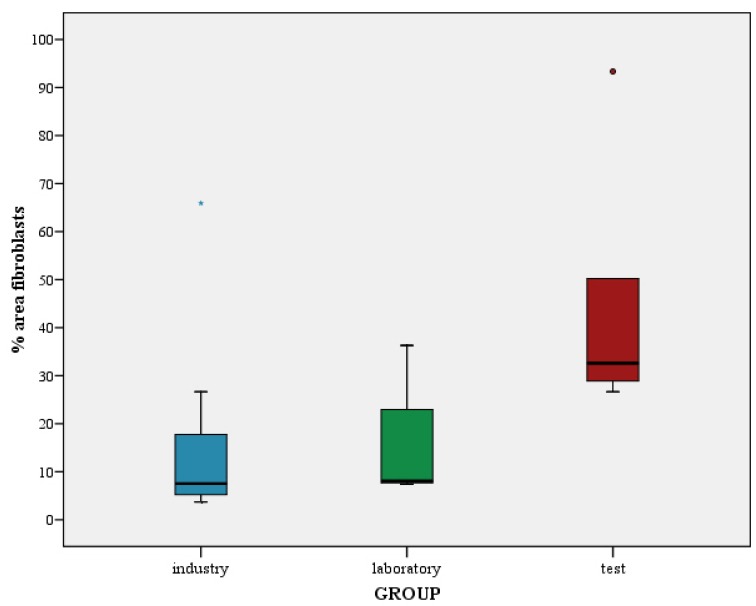
Distribution for the percentage of area occupied by cells per group.

The different cleaning processes yielded different levels of cell adhesion. From a descriptive perspective, abutments cleaned with plasma of Argon showed a marked higher cell adhesion than laboratory and industry abutments (Fig. 3). The mean percentages of area occupied by cells were 17.6 ± 22.7%, 16.5 ± 12.9% and 46.3 ± 27.9% for G1, G2 and G3 respectively.  Table 1 shows the descriptive statistics for the percentage of area occupied by cells.

**Table 1:**
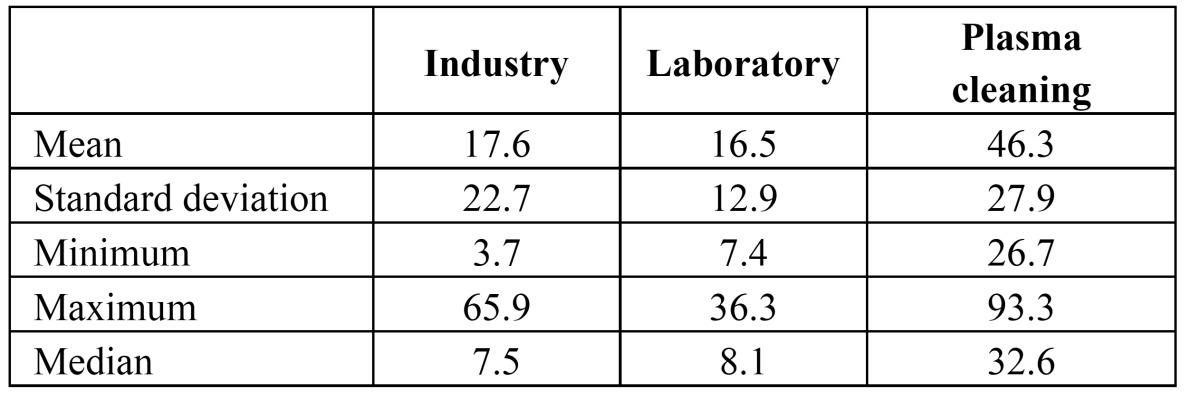
Descriptive statistics for the area of the abutment surface occupied by cells (%).

The comparative analysis yielded differences among the 3 study groups that were close to statistical significance (p = 0.052, K-W test). Differences were significant between G1 and G3 (p=0.030, test M-W), close to significant between G2 and G3 (p=0.056, M-W test), and non-significant between G1 and G2 (p=0.530, M-W test). For a Mann-Whitney test, using an alpha value set at 0.05 and considering a size effect of 0.8, statistical powers of 0.22 and 0.19 were calculated to detect differences in the percentage of area occupied by cells between G1-G3 and G2-G3 respectively. However, the size of the differences was greater than assumed and permitted finding statistically relevant results.

Regarding the presence/absence of cells, no cell could be detected in 2 out of 18 samples (1 from G1 and 1 from G2). The proportion of samples presenting cells was homogeneous in the 3 groups (p=1.000; Fisher test). In all cases adhered cells presented a similar flattened aspect (Fig. 1); in 2 cases (one from G1 and another from G2) cells were less efficiently adhered and in one of these 2 cases (from G1) cells presented filipodia. Three cases showed contamination with cocobacteria (one from each group).

## Discussion

The long-term success of dental implants depends not only on the integrity of osseointegration but also on the health of the peri-implant epithelium and the quality of attachment of the connective tissue to the supracrestal surface of implant components (1). It is hypothesized that achieving a tight soft tissue sealing around the transmucosal implant component might be useful to prevent epithelial downgrowth and hence infection, crestal bone loss and further soft tissue recession (12). Therefore, many efforts have been made to improve the sealing of the implant surface through the soft tissues.

It has been proven that the properties of the material placed in contact with the soft tissues have a decisive importance in the quality of the mucosal attachment. The chemical composition (13,14) and the surface topography (15) of the supracrestal portion of the implant are probably the two best studied properties. Abrahamsson et al. (14) claim that the abutment material may play an important role in the prevention of crestal bone and soft tissue recession. Controversially, Linkevicius and Apse (16) reviewed the literature and concluded that there was no evidence that titanium abutments perform better in maintaining stable peri-implant tissues, compared to other materials.

Similarly, a large number of processes are available to alter surface topography of titanium implants, such as machi-ning/micromachining, particle blasting, Ti plasma spraying, HA plasma spraying, chemical/electrochemical etching, or anodization. *In vitro* experiments suggest that human gingival epithelial cells attach and spread more readily on polished and etched titanium than on rougher surfaces (17). Similarly, smooth or finely grooved surfaces show higher human gingival fibroblast adhesion in *in vitro* studies (18,19). On the contrary, *in vivo* studies do not reflect this effect of surface roughness on cell adhesion. Abrahamsson et al. (15) and Glauser et al. (20) studied, in animals and humans respectively, the composition of the soft tissue barrier that formed in contact with abutments with smooth or rough surfaces and found that it was not influenced by the surface roughness.

Other properties, such as surface contamination and wettability, have also been shown to influence the behavior of soft tissue cells. *In vitro* studies have demonstrated that contamination of titanium surfaces reduces fibroblast cell attachment and spreading (8,21). Similarly, *in vitro* studies have related higher fibroblast adhesion and proliferation with increasing material surface wettability (22,23).

The use of plasma cleaning treatment on synthetic polymers has been shown to be effective to both clean surfaces from contaminants and to dramatically increase their wettability, thus enhancing their attractiveness to cells (24,25). Two recent studies demonstrated that Argon plasma has a double effect on titanium abutments: pollutions removal and increase of cell adhesion (9,10). Treatment with Argon plasma has been shown to enhance osteoblast adhesion, early bone formation and osseointegration of titanium implants (26-28). Argon plasma has also positive effects on soft tissue adhesion around implants although the evidence is more limited. Improvements in fibroblast adhesion in relation with surface cleanliness and wettability have been shown with metal surfaces; Baier et al. (29) tested plasma of Argon on germanium and Co–Cr–Mo implants. Coelho et al. (27) in a study on dogs with the primary objective of studying the effect of plasma on osseointegration, reported an improved interaction between connective tissue and plasma-treated titanium implants after 1 week. No clinical study had previously assessed the influence of plasma of Argon on the soft tissue attachment around implant components. The present study aimed at evaluating early soft tissue cell adhesion to titanium abutments subjected to different cleaning procedures.

Within the limits of the study, results suggest a better adhesion of soft tissue cell to titanium abutments cleaned by plasma of Argon than to those inserted as they come from the industry or cleaned by steam after laboratory customization. The reported preliminary results encourage further investigation of this technology but should, for now, be evaluated with great caution. The clinical significance of this study is limited by the small sample size and the short follow-up. Moreover, a 7-day period is not long enough and a cell adhesion analysis not relevant enough to have an accurate understanding of the effect of plasma of Argon on soft tissue attachment. The composition of the protein film and the orientation of the molecules that are absorbed on the titanium surface should be additionally evaluated in future studies to give a better understanding of the impact of plasma treatment on early soft tissue healing.

Additionally, although it was demonstrated to be efficient, analyzing images at 500x magnification might encumber a perfect recognition of cells or pollutants.

Therefore, the authors suggest that the investigation needs to be duplicated with a larger patient sample, a longer follow-up and additional techniques to analyze soft tissue adhesion.
